# Allosan in the Treatment of Gonorrhœa

**Published:** 1911-08-26

**Authors:** Alfred Stanhope Dawson


### THE GENERAL PRACTITIONER'S COLUMN.

### ALLOSAN IN THE TREATMENT OF GONORRHŒA.

By ALFRED STANHOPE DAWSON, L.R.C.P., L.R.C.S., L.M.Edin.

Gonorrhœa is one of the troubles in which it is clearly of great importance to the sufferer that the remedy employed should not disclose to others, by odour of breath or secretion, the nature of his complaint. I have found an ideal remedial agent in allosan (Zimmer's). This is the allophanic acid ester of santalol; it displays the action of santalol to its fullest extent, but is in solid form (7½ grain tablets or powder), odourless and tasteless. It may be administered in doses of 15 grains three or four times daily, either alone or assisted with injections of protargol, nizin, or other suitable astringents or bactericides.

### Some Points in its Favour.

My professional experience concerning this portable and unobtrusive remedy has been so favourable that I think it opportune to bring it to the notice of my colleagues. Allosan marks a very distinct advance in gonorrhœa treatment and puts the routine remedies (balsam copaiba, sandalwood oil preparations, etc.), quite into the shade. I have used it very extensively in bladder, prostate and urethral inflammation, irritation, strangury, tenesmus, etc., among my cases being many of long standing where patients had failed to obtain benefit from a course of the ordinary remedial agents. Given in doses of two 7½ grain tablets three or four times daily, steadily continued for a few weeks, allosan has never failed to give complete satisfaction, and in many cases conspicuous improvement was observed in the very beginning of the treatment. There is no doubt that the convenient form of this antigonorrhœic, as well as the obvious advantages of tastelessness and odourlessness, greatly assist the physician in carrying the treatment to a successful issue, seeing that patients will carry a bottle of tablets in their pocket and take a remedy which does not at once disclose their condition, whilst the bother, and certainty of exposure, connected with bottles of copaiba or other mixtures, sandalwood oil capsules, etc., are in a great many cases sufficient reasons for a patient not to take what is prescribed for him. It is therefore not surprising that allosan has found many advocates.

The following may serve as typical cases amongst those treated by me with allosan during the last twelve months:—

1. A. T., œdema of prepuce, redness and considerable rawness and excoriation of urethral orifice; pain on pressure for a considerable distance along urethra, with slight enlargement of glands of both groins; watery but slightly viscid discharge. I put patient on allosan tablets from which he derived some immediate benefit; encouraged by this he continued the treatment and within a fortnight irritation and soreness abated. After one month he expressed himself as much improved. His perseverance was stimulated by his ameliorated condition; he soon convalesced and is now quite free from his trouble. Patient expressed his thanks to me for bringing so admirable a remedy to his notice, and there is no doubt that the relief obtained has strengthened his faith in drug treatment of gonorrhœa; owing to failure of all other treatment he had begun to despair of success. The course of this case was convincing of the efficacy of allosan, and my experience has since been repeated in a great number of similar cases.

2. J. C., an elderly man; confessed to repeated previous attacks of gonorrhœa. Constant desire to micturate, considerable amount of strangury. Fifteen grains allosan every four hours soon gave relief, and but for obstinate constipation which was habitual in his case and possibly retarded the allosan action to some extent, would have improved more rapidly. Perseverance with two allosan tablets every four hours, however, brought the usual good results within a fortnight, and in a month from the beginning of the treatment the patient was practically well.

3. F. D., discharge of glycerine-like consistence, inability to retain urine completely, with tenesmus and splitting of stream, painful emission of urine due either to irregular congestion or thickening of urethral mucous membrane, or to some slight stricture. He was given the usual dose of 15 grains allosan thrice daily, and in a fortnight expressed himself as improved but as yet complained of a few beads of viscid discharge. In a month he passed water with only slight irritation, and in six weeks had no discharge nor any sense of discomfort.

These cases may be taken as typical of the many of which I have retained notes; whether administered in powder or tablet form, the results have been very gratifying, and enable me to say emphatically that this new santalol derivative is of high therapeutic value in all cases of urethritis, acute and chronic, simple and complicated by prostatitis, cystitis, or chronic irritation of the urinary tract from kidney to urethral orifice. It diminishes discharge, strangury and tenesmus, and perseverance with it will in most cases lead to improvement and cure. I repeat that I owe much to this addition to the group of antigonorrhœics and that I use it invariably, either alone or in combination with injections, according to indications.

